# Rabies prophylaxis after an animal attack that caused a ruptured eye and traumatic cataract: a case report

**DOI:** 10.4076/1757-1626-2-9192

**Published:** 2009-09-15

**Authors:** Mike P Holzer, Kerry D Solomon

**Affiliations:** 1 Storm Eye Institute, Medical University of South Carolina, Ashley Avenue, Charleston SC, 29425, USA; 2 Department of Ophthalmology, University of Heidelberg, Im Neuenheimer Feld 400, 69120 Heidelberg, Germany

## Abstract

**Introduction:**

We report on a patient with an animal bite eye injury, his surgical treatment and proper rabies immunoglobulin administration.

**Case presentation:**

A 33-year-old Turkey hunter was attacked by a bobcat and his injuries included a ruptured globe with corneal laceration, two iris sphincter tears, and a ruptured anterior capsule with a traumatic cataract. Rabies vaccination was started, primary closure of the corneal laceration, an anterior chamber washout and one week later cataract surgery were performed. Three months postoperatively he achieved an uncorrected visual acuity of 20/50 and a best corrected visual acuity of 20/20.

**Conclusion:**

Bobcat attacks on humans are very rare and extremely suspicious for rabies infection of the animal. Ophthalmologists need to be aware of the importance of immediate and appropriate post exposure rabies vaccination. Proper rabies immunoglobulin administration in the setting of globe injuries is challenging and we report on the Center for Disease Control and Prevention recommendations for globe injuries.

## Introduction

Animal bites and scratches to the eye and ocular adnexa may result in severe injuries and are a significant medical problem. The vast majority of these bites are from pets such as dogs and cats [1]. In more than 80% of cases, the animal is known to the victim and injury usually occurs during voluntary interaction with the pet. In an epidemiologic review of 332 animal bite injuries Kizer found *Pasteurella multocida* to be the most common pathogen cultured with 50% and 80% culture-positive rates for dog and cat bite scratches, respectively [2]. Injuries with wild animals are much rarer, however, and can be even more serious than with pets, and infections like tetanus, rabies or wound botulism are possible. Rabies is caused by an infection with a rhabdovirus, which is transmitted mainly by saliva from infected mammals. The virus enters the central nervous system of the host and causes an encephalomyelitis that is, in almost all cases, lethal. The incubation period of the virus ranges between 21 and 60 days, depending on the size of the inoculum, the severity of laceration, and the distance of the injury from the brain [3]. Prodromal symptoms of the disease are fever, malaise, headache and cough and, later on, brainstem dysfunction with diplopia, irregular dilated pupils, excitation, facial palsy, coma, and finally resulting in death. Between 1980 and 1996, 32 cases of human rabies were reported in the United States, out of which seven had a definite exposure history [4]. Wild animals represent the vast majority of cases of animal rabies in the United States [5]. We report here a unique case of a bobcat attack on a hunter in the wilderness causing severe ocular injuries, demonstrate the surgical treatment and discuss problems that might occur with appropriate rabies immunization after eye injuries.

## Case presentation

**Figure 1:**
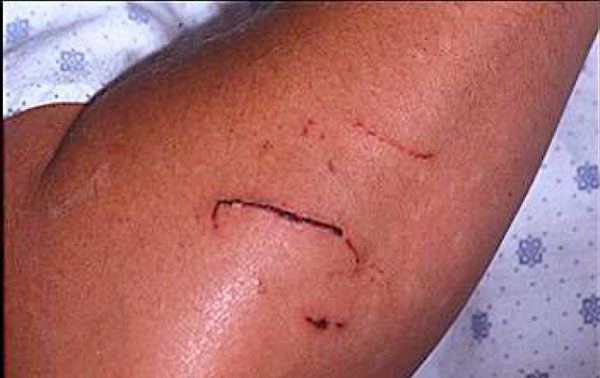
**Following the bobcat attack the patient had scratches on his right arm and on his face**. Rabies immunization was administered around the scratches on his arm.

**Figure 2:**
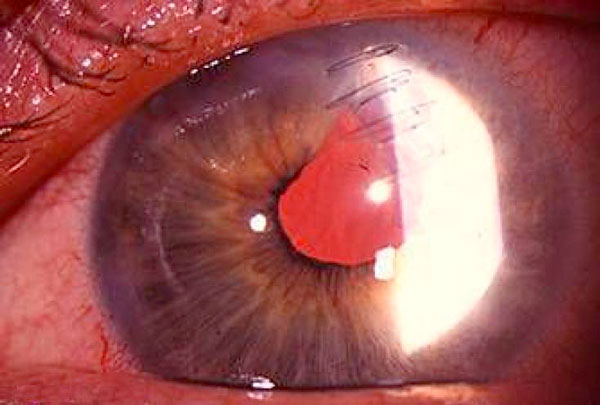
**Slit lamp photograph of the eye, one day after traumatic cataract removal and intraocular lens implantation**. The intraocular lens was centered with a best corrected visual acuity of 20/30 and the corneal laceration was closed without any leakage (Seidel negative).

A 33-year-old Turkey hunter was attacked by a bobcat (Lynx rufus). His injuries included a ruptured left eye with a 5 mm corneal laceration superior temporal, two iris sphincter tears at the area where the cornea was injured, and a ruptured anterior capsule with a traumatic cataract [6]. A B-scan ultrasound showed mild vitreous debris but no retinal detachment. The visual acuity of his left eye was at counting fingers at a distance of two feet. The right eye was not injured and in normal condition. He had no medical problems or history of any eye diseases or trauma and he had several scratches on his right arm and face (Figure 1). The patient reportedly was vaccinated against tetanus but not against rabies. Due to the aggressive behavior of the animal, rabies could not be ruled out but could also not be confirmed and therefore, rabies vaccination as well as a tetanus booster was administered immediately. The Center for Disease Control and Prevention (CDC, Atlanta, GA, USA) was contacted to get recommendations on how to administer the passive antibody and the vaccination for post exposure prophylaxis. The recommendation consisted of an immediate and thorough washing of all bite wounds and scratches with soap and water and a virucidal agent such as a povidone-iodine solution irrigation. This was performed for all his facial injuries and the scratches on his arm. The CDC also recommended, if anatomically feasible, to infiltrate the full immunoglobulin dose around the wound and to administer any remaining volume intra muscularly (IM) at an anatomical site distant from the vaccine administration. This was, in this specific case, not feasible and therefore immunoglobulin was administered at a dosage of 20 international units (IU) per kg of body weight into his arm around the scratches and vaccination was started at the same time with IM injection in his deltoid muscle and repeated on days 3, 7, 14 and 28. This was followed by primary closure of the corneal laceration and an anterior chamber washout. The patient was on a topical fluoroquinolone antibiotic, a steroid (prednisolone acetate 1%) and cyclopentolate hydrochloride 1% to maintain iris motility. Additionally, oral antibiotic coverage with 500 mg ciprofloxacin hydrochloride twice per day was started. One week later, his cornea was clear, the laceration did not leak, and the intraocular pressure was normal. No signs of ocular infection were noted and the decision was made to remove the traumatic cataract, repair the pupil tears and implant an intraocular lens (IOL). An AcrySof^®^ MA60AC IOL (Alcon Laboratories, Inc., Fort Worth, TX, USA) was implanted symmetrically into the capsular bag and oriented 90° away from the anterior capsule extension (Figure 2). Three months after the surgery all remaining sutures were removed and the patient enjoyed a best-corrected visual acuity of 20/20.

## Discussion

Animal bite injuries occur in the United States in more than a million cases per year, a number that probably only represents 25% to 50% of all incidents [2]. Herman et al report even more cases with an estimate of 3.5 million cases per year [1]. Twenty percent of dog bites and cat scratches or bites involve the head and neck area. Of all cat injuries to the head and neck, 60% involve the globe or ocular adnexa and 40% of the patients sustain corneal abrasions. This leads to the estimate of approximately 300,000 globe and ocular adnexa injuries due to animal bites in the United States every year making it an important socioeconomic and health care problem. The case reported in this article shows the potential complexity of such cases. The fact that the patient was attacked by a wild animal arouses suspicion that the animal may have a rabies infection. Rabies is a viral infection transmitted in the saliva of infected mammals. The virus enters the central nervous system of the host, causing an encephalomyelitis that is almost always fatal. As reported by the CDC, rabies among wildlife occurs throughout the continental United States and the animals most often infected include raccoons, skunks and bats; only Hawaii seems to remain consistently rabies-free [7]. The attacking animal in this case report was a bobcat, which is also known as a wildcat or bay lynx and its distribution is all over the continental United States and the Eastern border of Canada. In the case of human attacks an immediate consultation with a doctor is recommended in order to start appropriate post exposure rabies prophylaxis. The latest recommendation by the CDC for cases of not previously vaccinated people states that firstly the wound should be thoroughly cleansed with soap, water and, if available, povidone-iodine, followed by passive immunization which should be started immediately afterwards with 20 IU of rabies immune globulin per kg of body weight. This should be infiltrated around the wound and any remaining volume should be administered intramuscularly at an anatomic site distant from the wound. In the reported case the wound was not infiltrated with passive immunoglobulin due to the unknown toxicity to intraocular tissue. Infiltration of lid lacerations on the other hand seems to be quite reasonable for rabies prophylaxis. The vaccine to induce an active immune response with neutralizing antibodies should be injected into the deltoid muscle at a dosage of 1.0 ml on day 0 (first day of vaccination), then on days 3, 7, 14, and 28. The vaccine is available as human diploid cell vaccine (HDCV), purified chick embryo cell (PCEC) vaccine and rabies vaccine adsorbed (RVA). If a person has previously been vaccinated, wound cleansing should be performed as described above and the vaccine for active immune response should be administered into the deltoid muscle in the dosage of 1.0 ml, however, only on day 0 and day 3. Rabies immune globulin (RIG) for passive immunization should not be administered [7]. There is no cure for rabies once symptoms begin and the disease is always fatal; the postexposure prophylaxis can only prevent the disease if given before symptoms start. Therefore, immediate prophylaxis in suspicious cases is essential. However, even postexposure prophylaxis in rare cases cannot prevent the disease. Tabbara and Al-Omar reported on two patients with eyelid lacerations after an attack by a rabid desert fox and one of the patients died despite prophylactic treatment [8]. They attributed the death to the size of the inoculum and the proximity of the laceration to the cranial nerves. Haltia et al described the ocular pathology of rabies when they reported the first European case of human bat-borne rabies [8]. They found granules with rabies virus antigen in the cytoplasm of retinal ganglion cells as well as in the patient's brain. Additionally, they detected glial fibrillary acidic protein (GFAP) in Müller cells at the ora serata. They postulate that this occurred as a response to retinitis and retinal vasculitis as observed in their case. Other possible ocular manifestations of rabies in humans include the cornea. Schneider reported in 1969 the cornea test to intra-vitally diagnose rabies [9]. He found that viral antigen could be detected via fluorescent antibody testing of corneal epithelial cell impressions. Normally the virological diagnosis of rabies is made in man or animals only at an autopsy by examining the brain [10]. The most common ways of rabies transmission in the United States are bat bites, which accounted for 21 out of 36 human cases of rabies diagnosed in the United States since 1980 [5,7]. Four cases of transmission through corneal transplants have been reported so far worldwide since 1980 with one case in the United States [11]. Other potential ways of transmission, besides animal bites, include breast feeding, transplacental, aerosol inhalation and the rabies virus can also be detected in tracheal and nasal secretions, tears, urinary sediment and more [12].

## Conclusion

In conclusion, the case reported shows the potential danger of animal attacks on humans and demonstrates subsequent important steps to treat ocular injuries and start proper rabies and tetanus immunization.

## Abbreviation

CDC: The Center for Disease Control and Prevention.

## Consent

Written informed consent was obtained retrospectively from the patient for publication of this case report and accompanying images. A copy of the written consent is available for review by the Editor-in-Chief of this journal.

## Competing interests

The authors declare that they have no competing interests.

## Authors' contributions

KDS performed the surgical treatments and follow-up of the patient. MPH reviewed the case and the literature and prepared the manuscript. Both authors read and approved the final manuscript.
